# Identifying Protein Interactions with Viral DNA Genomes during Virus Infection

**DOI:** 10.3390/v16060845

**Published:** 2024-05-25

**Authors:** Jessica E. Packard, Namrata Kumar, Matthew D. Weitzman, Jill A. Dembowski

**Affiliations:** 1 Department of Biological Sciences, School of Science and Engineering, Duquesne University, Pittsburgh, PA 15282, USA; 2 Department of Pathology and Laboratory Medicine, Perelman School of Medicine, University of Pennsylvania, Philadelphia, PA 19104, USA; 3 Division of Protective Immunity, Children’s Hospital of Philadelphia, Philadelphia, PA 19104, USA

**Keywords:** DNA–protein interaction, DNA virus, DNA replication, gene transcription, DNA repair, viral replication compartment, nucleus

## Abstract

Viruses exploit the host cell machinery to enable infection and propagation. This review discusses the complex landscape of DNA virus–host interactions, focusing primarily on herpesviruses and adenoviruses, which replicate in the nucleus of infected cells, and vaccinia virus, which replicates in the cytoplasm. We discuss experimental approaches used to discover and validate interactions of host proteins with viral genomes and how these interactions impact processes that occur during infection, including the host DNA damage response and viral genome replication, repair, and transcription. We highlight the current state of knowledge regarding virus–host protein interactions and also outline emerging areas and future directions for research.

## 1. Introduction

Viruses are ubiquitous pathogens that manipulate the host cell to create an environment favorable for infection. Viruses containing genomes of single-stranded and double-stranded deoxyribonucleic acid (DNA) typically replicate in the host cell nucleus, with the exception of poxviruses that replicate in cytoplasmic compartments [1,2]. Virus particles with DNA genomes can undergo a complex process of disassembly as their genomes are transported to the nucleus [3,4]. Nuclear entry initiates an early stage of infection that involves the transcription of genes that are essential for viral DNA replication. The late stage of infection encompasses the production of structural and capsid proteins that are required for efficient viral packaging and progeny production. DNA viruses have limited coding capacity and generally replicate their genomes in highly crowded nuclear environments. Therefore, the outcome of infection will be determined by cellular factors that interact with viral genomes during infection.

Viruses co-opt cellular machineries to favor viral processes, including viral gene transcription and DNA replication. Viruses must also counteract a number of host processes that act to restrict infection. Investigating virus–host interactions provides an understanding of viral pathogenesis as well as insights into novel roles that cellular proteins play in the context of virus infections. In this review, we focus on current experimental proteomics techniques utilized to examine interactions of cellular proteins that take place specifically on viral DNA genomes. We mainly concentrate our discussion on *Herpesviridae* and *Adenoviridae*, which form nuclear sub-compartments during infection, as well as *Poxviridae*, which form sub-compartments in the cytoplasm. We also discuss how three-dimensional (3D) genome technologies can be applied to examine changes in nuclear architecture and cellular networks during infection as viral and cellular proteins associate with both virus and host genomes. We highlight emerging areas and future directions for research in this crucial area of understanding virus–host interactions at the level of genome interactions.

## 2. Overview of Approaches to Study Protein–DNA Interactions in Cells

Protein–DNA interactions are dynamic, highly regulated, and drive fundamental processes, including transcription, DNA replication, recombination, and repair. Specific interactions between a viral DNA sequence and a particular viral protein can be studied using biochemical approaches such as electrophoretic mobility shift (EMSA) assays [5] or genetic approaches such as one-hybrid reporter systems [6,7]. Biochemical approaches can also identify factors that are associated with a viral protein by affinity purification from cell extracts [8] and can map the precise regions of interaction by DNA footprinting [9]. Proteins that bind to viral genomes can be used to isolate additional factors bound to the same DNA molecules or associated factors by immunoprecipitation and mass spectrometry (IP-MS) [10]. These classic approaches have been used successfully to identify cellular proteins that are associated with viral DNA genomes [11,12] and have been complemented recently by new techniques that isolate proteins directly interacting with viral genomes within infected cells. Techniques to study protein–DNA interactions in cells each have their own advantages and limitations, and these approaches are discussed below. In many cases, the use of a combination of approaches is necessary to understand interactions comprehensively within cells.

### 2.1. Chromatin Immunoprecipitation (ChIP) and Cleavage under Targets and Release Using Nuclease (CUT&RUN)

**Figure 1:**
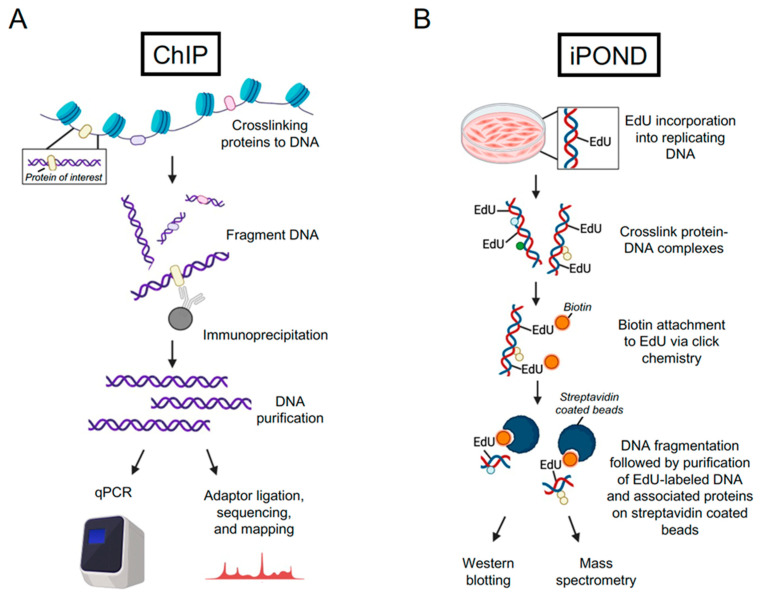
Techniques to identify viral genome-associated factors include ChIP and iPOND. (**A**) Outline of the ChIP approach to identify the DNA binding sites of a viral or cellular protein of interest. (**B**) Outline of the iPOND approach to identify proteins associated with EdU-labeled nascent DNA. Modifications and alternative approaches to studying viral DNA–protein interactions are further discussed in the text. Created with BioRender.com (accessed on 8 April 2024).

Binding of proteins to eukaryotic genomes can be assessed by chromatin immunoprecipitation (ChIP), and when combined with sequencing, it can provide a global map of DNA binding sites across the genome. ChIP is typically used to monitor transcriptional regulation, including binding to DNA by histones, transcription factors, and RNA polymerase [13]. This approach requires formaldehyde crosslinking to stabilize protein interactions, followed by fragmentation of genomic DNA by sonication or micrococcal nuclease (MNase) digestion, enrichment of the desired protein using a specific antibody, and detection of the crosslinked DNA using high-throughput sequencing (ChIP-seq) [14] or quantitative polymerase chain reaction (ChIP-qPCR) [15] (Figure 1A). The inherent nature of this assay is that it relies on an antibody to isolate the protein of interest and bound DNA from cells. Specific antibodies are not always available, and tagging the protein of interest can disrupt normal DNA binding. Substrate identification by qPCR requires prior knowledge of DNA binding sites for the design of primers for detection, which can introduce potential bias to this approach. The advantages of ChIP-qPCR include that it is inexpensive and relatively quick to complete. The advantages of ChIP-seq include the ability to monitor protein–DNA interactions at a genome-wide level without prior knowledge of the DNA binding sequence. For both ChIP-seq and ChIP-qPCR, the crosslinking step may enable transient interactions to be captured. ChIP-seq data can be used to identify consensus DNA binding motifs of DNA-binding proteins and to correlate genome binding with differential gene expression and transcription regulation.

An alternative to ChIP-seq to map genome-wide protein–DNA interactions is cleavage under targets and release using nuclease (CUT&RUN) [16]. This approach utilizes native conditions and was developed based on chromatin immunocleavage (ChIC) [17]. To carry out CUT&RUN, nuclei are captured on lectin-coated magnetic beads and incubated with an antibody specific for the target protein, followed by incubation with protein A-MNase. Calcium is then added to activate the cleavage reaction, which is subsequently stopped by chelation of calcium. The samples are then centrifuged to separate the nuclei from the supernatant, which contains released protein–DNA complexes. DNA is extracted and used for adaptor ligation and library preparation for downstream sequencing analysis. One advantage to CUT&RUN is that it produces low background and, therefore, requires less sequencing depth compared to ChIP-seq. Therefore, it can be modified for analysis of protein binding in single cells [18]. Another adaptation of CUT&RUN that uses a fusion of protein A and Tn5 transposase to fragment DNA and ligate adaptors for PCR enrichment is cleavage under targets and tagmentation (CUT&Tag) [19]. Note that because native conditions are used for CUT&RUN and related techniques, antibodies that are validated for ChIP may not work for these assays.

### 2.2. Unbiased Techniques to Identify Proteins Associated with Nascent DNA

Unbiased approaches to identify DNA interacting proteins include DNA immunoprecipitation assays such as isolation of proteins on nascent DNA (iPOND) [20], DNA-mediated chromatin pull down (Dm-ChP) [21], and nascent chromatin capture (NCC) [22]. For the iPOND approach, cells are incubated with 5-ethynyl-2’-deoxyuridine (EdU), which becomes incorporated into replicating DNA [20,23] (Figure 1B). Formaldehyde is then used to crosslink protein–DNA complexes, cells are permeabilized, biotin is covalently attached to EdU via click chemistry, cells are lysed and DNA is fragmented by sonication, and EdU-labeled nascent DNA and associated proteins are isolated on streptavidin-coated beads. This technique, coupled with MS, allows an unbiased analysis of proteins associated with replication forks, replicating DNA, or replicated DNA [24,25]. The iPOND has been successfully used to study replication fork dynamics of replisome proteins; protein recruitment to damaged, stalled, or collapsed replication forks; and chromatin dynamics [20,23,25]. Similar approaches to iPOND include Dm-ChP [21] and NCC [22]. The main differences from iPOND are that Dm-ChP combines EdU labeling of nascent DNA with biotin-TEG azide attachment via click chemistry, and for NCC, replicating DNA is selectively labeled with biotin-dUTP. Each of these approaches is followed by elution in Laemmli sample buffer at a high temperature to reverse formaldehyde crosslinking.

Use of formaldehyde crosslinking to preserve DNA–protein binding during purification can interfere with protein identification via immunoblotting and mass spectrometry (MS). To simplify MS analysis, Sirbu et al. proposed native iPOND (niPOND) that does not use formaldehyde crosslinking [23]. In addition, Leung et al. designed and validated accelerated native iPOND (aniPOND) to improve the efficiency of the iPOND procedure and subsequent MS analysis [26]. For aniPOND, after EdU labeling of replication forks, cell lysis, and nuclei harvesting are performed in the dish simultaneously, which reduces sample manipulation and loss. The click reaction to biotinylate EdU-labeled DNA is carried out in intact nuclei, and rather than formaldehyde crosslinking, aniPOND uses mild detergent conditions for nuclear lysis and native conditions for purification of protein–DNA complexes. These adaptations were performed in adherent cells. Furthermore, Wiest and Tomkinson optimized the conditions to perform iPOND and aniPOND in suspension cells [27].

Another adaptation of the iPOND protocol is mapping in vivo nascent chromatin with EdU and sequencing (MINCE-seq) [28]. Like iPOND, replicating DNA is labeled with EdU, followed by a click reaction to tag the labeled DNA with biotin. However, after the click reaction, DNA that is not bound by proteins is digested with MNase. Next, the proteins are degraded, and the regions of DNA that were protected from nuclease digestion are purified on streptavidin-coated beads. Isolated DNA is then sequenced, and nucleosome and transcription factor binding on nascent DNA can be deduced based on the patterns of MNase protection.

### 2.3. Validation of Protein–DNA Interactions

The techniques described above use isolation of proteins or DNA to identify potential protein–DNA interactions. iPOND and its adaptations have been powerful tools for studying DNA–protein interactions during cellular DNA replication, including replisome dynamics, chromatin assembly on nascent DNA, and protein recruitment to damaged replication forks. These interactions can be further validated in vitro and in cells. Protein immunoprecipitation (IP) with specific antibodies can be used to identify protein interaction partners and protein complexes of known DNA-binding proteins. However, protein IP does not reveal the sub-cellular location of protein interactions within the cell or on DNA. On the other hand, immunofluorescence (IF) imaging can be used to label and detect proteins and thus provide spatial information regarding where proteins are localized in the cell and their position relative to each other. In addition, EdU-labeled DNA can be covalently attached to a fluorophore via a click reaction to determine where proteins localize relative to labeled DNA [29]. However, much like ChIP approaches, protein IP and IF are antibody-specific techniques and require the use of a validated antibody and prior knowledge of the protein under investigation.

## 3. iPOND as a Tool to Identify Host Factors Associated with Viral Genomes

**Figure 2:**
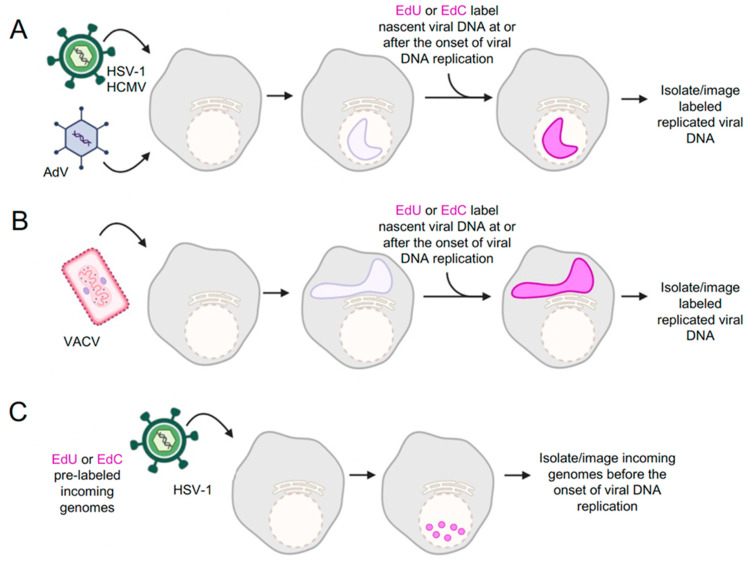
iPOND and aniPOND have been used to investigate protein association with different populations of viral DNA. (**A**) HSV-1, HCMV, and AdV form nuclear replication compartments. Viral DNA can be labeled by incorporation of EdU, EdC, or other nucleoside analog into nascent viral DNA. (**B**) VACV forms cytoplasmic factories where nascent viral DNA can be labeled with nucleoside analogs. (**C**) Cells can be infected with infectious viruses containing EdC or EdU-labeled genomes. This enables the isolation of viral DNA and investigation of processes that occur before or in the absence of viral DNA replication. Not shown: The use of pulse labeling and viral mutants can also provide information about temporal and spatial control of infection. In addition, discrete populations of viral DNA can be covalently attached to a fluorophore by click chemistry to visualize by fluorescence microscopy. Created with BioRender.com (accessed on 8 April 2024). Detailed protocols provided in [35].

Techniques developed for identifying and analyzing protein–DNA interactions have been adapted for studying virus–host interactions. Here, we will focus on recent studies that have utilized iPOND or aniPOND for the unbiased identification of viral genome-associated proteins. Conceptually, iPOND can be adapted to study protein binding to any population of DNA that can be selectively labeled with EdU or other “clickable” nucleoside analogs such as 5-ethynyl-2’-deoxycytidine (EdC) (Figure 2). A challenge for a complete understanding of nuclear processes active on viral genomes throughout the infectious cycle is knowledge of which cellular proteins associate with the viral genome at each stage of infection. Dembowski and DeLuca adapted iPOND and aniPOND to isolate herpes simplex virus type 1 (HSV-1) genomes at different stages of infection and identified viral and cellular proteins associated with replicated and replicating viral DNA (Figure 2A) [30]. To maximize EdU incorporation into viral DNA, a mutant virus defective for expression of the viral uracil glycosylase (UL2) and dUTPase (UL52) was used for infection. EdU was incorporated into replicating viral DNA at time points after the onset of viral DNA replication, between 4–6, 6–8, and 8–12 h post infection (hpi). A key experimental detail was an infection of cells that were in G_0_ in order to ensure that cellular DNA would not be replicated and labeled with EdU. In this study, iPOND and aniPOND were carried out, followed by MS, to identify proteins bound to HSV-1 DNA after the onset of DNA replication. Overlapping and unique sets of proteins were identified by aniPOND and iPOND. Although aniPOND was more sensitive, transient interactions could be captured by iPOND. Later studies demonstrated that EdU and EdC can be effectively incorporated into HSV-1 DNA in the presence of UL2 and UL50 and that these mutations are not necessary for iPOND or aniPOND analysis of the HSV-1 genome [31,32]. For the first time, these experiments presented a comprehensive view of the proteins that are associated with HSV-1 genomes, providing new insight into cellular factors involved in HSV-1 infection.

The application of iPOND-MS to a wider range of viruses was demonstrated by Reyes et al., who performed iPOND experiments with adenovirus type 5 (Ad5), HSV-1, and vaccinia virus (VACV) (Figure 2A,B) [31]. EdU was incorporated for 15 min at the peak of viral DNA synthesis for each virus (Ad5 at 24 hpi, HSV-1 at 8 hpi, and VACV at 6 hpi), and proteins associated with replicating viral DNA were identified. One notable observation, consistent with [30], was that cellular DNA polymerases were mostly absent from replicating viral DNA. The isolation of known viral replication proteins on nascent labeled viral genomes is further validation of iPOND for each of these viral systems. Each virus encodes its own viral DNA polymerase, and these were preferentially enriched on replicating viral DNA more than the cellular polymerases. However, other cellular DNA replication and repair proteins were enriched on viral DNA, suggesting that select cellular factors contribute to viral DNA replication and repair. Senkevich et al. also used iPOND coupled with MS to identify VACV replisome and transcriptome proteins (Figure 2B) [33]. Many of the viral and cellular proteins found to be associated with VACV DNA overlapped with the findings of Reyes et al. [31]. Together, consistent iPOND-MS results for HSV-1 and VACV from different labs demonstrate that iPOND is a highly reproducible approach to investigating virus–host interactions during infection.

In addition to the investigation of replicated viral DNA within replication compartments [30], aniPOND and iPOND have been used to study less abundant populations of viral DNA, including replication forks [31,32] and infecting viral genomes before and after the onset of viral DNA replication [34]. To study replication fork dynamics, replicating viral DNA was pulse-labeled with EdC and chased with deoxycytidine to investigate protein association during and post HSV-1 DNA replication [32]. In addition, to define protein interactions before the onset of viral DNA replication, cells were infected with HSV-1 virions containing viral genomes that were pre-labeled with EdC (Figure 2C) [34]. These studies have provided insight into protein dynamics at HSV-1 replication forks and on infecting viral DNA during the early stages of infection and demonstrate the feasibility of using iPOND and related approaches to study distinct populations of viral DNA.

iPOND and aniPOND have also been used to investigate viral mutants defective for HSV-1 immediate early gene products ICP4, ICP22, and ICP0 [34,36,37]. ICP4 is the major viral transcription factor, and viruses defective for ICP4 expression have strong defects in transcription factor recruitment to viral DNA [34]. ICP22 is important for viral transcription elongation, and viruses defective in ICP22 expression fail to recruit transcription elongation factors to viral DNA [36]. ICP0 is an E3 ubiquitin ligase that counteracts host intrinsic defenses to infection by targeting cellular proteins for proteasomal degradation [38,39]. Identification of proteins bound to ICP0 mutant viral genomes revealed novel antiviral factors, including the cellular protein Schlafen family member 5 (SLFN5), which are targets of the ICP0 ubiquitin ligase activity [37]. Taken together, the high sensitivity of viral iPOND approaches has enabled the comprehensive identification of cellular proteins associated with HSV-1 genomes throughout the course of infection. While these studies have generated extensive datasets of cellular proteins accumulating on viral DNA, significant work still needs to be conducted to validate the functional significance of many of these interactions.

iPOND and related techniques have also been modified to study infection of other DNA viruses, including Epstein–Barr virus (EBV), human cytomegalovirus (HCMV), and Kaposi’s sarcoma-associated herpesvirus (KSHV) [40,41,42]. Xu et al. utilized iPOND to identify cellular proteins that are upregulated at cellular replication forks to contribute to the ability of EBV to drive DNA replication and cell proliferation [40]. Manska and Rossetto demonstrated that virally encoded replication proteins and many host factors, including cellular replication, transcription, chromatin remodeling, repair, and RNA processing factors, are associated with the replicating HCMV genome [41]. Furthermore, Dabral et al. combined ChIP and iPOND to detect proteins that are associated with KSHV latency-associated nuclear antigen (LANA)-bound (EdU-labeled) replicated DNA [42]. Finally, Dybas et al. combined iPOND with proteomics to investigate viral remodeling of the host proteome and host factors associated with AdV genomes for both wild-type and mutant virus infections [43]. Together, these studies have established the iPOND-MS technique as a useful approach for studying virus–host interactions and identifying cellular regulators.

## 4. Cellular Proteins That Interact with Viral DNA Genomes

In addition to virally encoded proteins that are associated with viral DNA genomes, a plethora of host factors have been found to be associated with genomes of double-stranded DNA viruses. These proteins fall broadly into classifications of transcription factors, replication factors, DNA repair proteins, and antiviral restriction factors.

### 4.1. Transcription Factors

HSV-1, Ad5, and VACV all encode transcription factors that help to activate viral genes in a temporal cascade. During productive HSV-1 infection, viral genes are transcribed by host RNA polymerase II (Pol II) through a regulated cascade of immediate early (IE), early (E), and late (L) genes. IE genes are expressed without viral protein synthesis, largely through the actions of the viral tegument protein VP16 [44]. IE gene products include the major viral transcription factor ICP4, transcriptional regulator ICP22, and RNA-processing protein ICP27. IE gene products activate transcription of E genes, whose products are required for viral DNA replication [45,46]. Viral DNA replication is required for the expression of L genes, whose gene products are involved in the assembly of virions [47]. It is not completely understood how DNA replication and late gene transcription are coupled. The viral transcription regulators of HSV-1 that were found to associate with the viral genome via iPOND and aniPOND include VP16, ICP4, ICP22, and ICP27 [30,31]. These approaches have also demonstrated that cellular transcription factors are enriched on HSV-1 DNA. These include Pol II, transcription factor IID (TFIID), lysine-specific demethylase 1 (LSD1), the facilitates chromatin transcription (FACT) histone chaperone complex, the Mediator transcription coactivator complex, and RNA processing factors. Viral VP16 and ICP4 proteins function to recruit Pol II to immediate early and early/late genes, respectively. They also function to recruit cellular factors involved in transcription, including the TATA-binding protein (TBP), TFIID, and the Mediator complex, to aid in viral transcription initiation [48,49,50,51,52]. In the absence of ICP4, the recruitment of cellular TFIID, Mediator, and Pol II to viral DNA is significantly reduced [34]. Furthermore, ICP22 is important for the regulation of transcription elongation [53,54], and in the absence of ICP22 transcription elongation factors, including the FACT complex (SSRP1/Spt16), Spt5 and Spt6 have reduced association with viral DNA [36]. Taken together, iPOND data provide support for models regarding mechanisms by which viral proteins recruit host transcription machinery to viral DNA to regulate the temporal expression of viral genes.

Like HSV-1, the AdV genome is also transcribed by host Pol II. Gene expression switches from early to late in a tightly regulated manner. The early AdV genes are the first to turn on, and they include genes that encode proteins critical for transcription, as well as the viral replication machinery. Late genes are mostly transcribed following the onset of viral DNA replication. These include the major late transcriptional unit, which codes for capsid and packaging proteins [55]. An early viral transcription factor E1A that associates with the viral genome was identified by iPOND [31]. A conserved region of E1A, denoted CR3, activates early gene promoters as a result of an interaction with the host Mediator complex [56]. The iPOND-MS analysis identified TFII-I, a cellular transcription factor, to be deficient in replicated viral DNA during Ad5 infection [31]. Consistent with this observation, it was found that TFII-I is targeted for degradation by the viral E4orf3 protein. Viral factors E4orf3, along with E1b55K and E4orf6, are early gene products that counteract the intrinsic cellular defense against infection by targeting cellular proteins for degradation or relocalization [57]. Over the course of infection, TFII-I levels decrease when infected with wild-type Ad5, whereas levels remain high when infected with a mutant lacking the E4 region [31]. This demonstrates that viral factors that target host proteins to the proteosome indirectly contribute to the regulation of protein association with viral DNA. On the other hand, host nucleolar proteins involved in ribosomal RNA expression and processing were found to associate with Ad5 DNA. The host factor treacle ribosome biogenesis factor 1 (TCOF1), which regulates ribosomal DNA transcription and pre-ribosomal RNA processing, was found to associate with the Ad5 genome [31]. TCOF1 helps to recruit nucleolar factors to Ad5 DNA and is important for late viral gene expression and efficient viral DNA replication. These data indicate that iPOND can help to identify novel roles for cellular transcription factors in viral infection and reveal how AdV exploits host transcription factors to promote efficient viral transcription. Recruitment of cellular factors may be affected by viral proteins bound to viral genomes, as well as factors that manipulate the proteome but are not associated with viral DNA directly.

In contrast to both HSV-1 and AdV, the larger DNA genome of VACV replicates in the cytoplasm and encodes its own multi-subunit DNA-dependent RNA polymerase. Over 200 viral genes are transcribed in a temporal order, including early, intermediate, and late genes. The first class of genes that are transcribed are early genes, which include DNA replication factors, intermediate gene transcription factors, and factors that evade host defenses [58]. Transcription factors that activate transcription initiation of the early genes are brought into the cell in the virion. Intermediate transcription factors and DNA replication drive intermediate gene expression, which includes late gene transcription factors. Subsequently, late gene products include proteins involved in virion assembly. The four largest of the eight subunits of the viral RNA polymerase were recovered from nascent VACV DNA using iPOND [33]. In addition, the intermediate transcription factor proteins (A23, A8), the late transcription proteins (G8, A1, A2), the transcription elongation factor (G2), the capping enzyme (D1, D12), and the 2’ O-methyltransferase (J3) were identified among proteins that bind to the VACV genome during infection [33]. Four cellular transcription factors that were previously implicated in VACV transcription regulation were also found to be associated with viral DNA. These include Caprin 1, heterogenesis nuclear ribonucleoprotein A2/B1 (hnRNP A2/B1), RNA-binding motif protein 3 (RBM3), and Ras-GTPase activating protein–binding protein 1 (G3BP1) [33]. RNA-binding proteins hnRNP A2/B1 and RBM3 have both been identified to help activate late gene expression [59]. The role that Caprin 1 and G3BP1 play in VACV transcription is not well understood. However, both transcription factors were shown to colocalize with viral mRNA [60]. Taken together, the viral iPOND data indicate that VACV transcription is mediated by mostly viral factors, which is consistent with previous observations.

### 4.2. Replication Factors

The iPOND-MS technique has successfully been used to study DNA viruses that replicate in the nucleus of host cells (e.g., AdV and HSV-1) (Figure 2A) as well DNA viruses that replicate in the cytoplasm (e.g., VAVC) (Figure 2B). All three viruses express genes that encode key proteins of the viral replication machinery. The smaller AdV genome encodes a DNA polymerase (Ad Pol, E2B), a pre-terminal protein (pTP), and a DNA-binding protein (DBP, E2A), which are essential for viral DNA replication [55]. For HSV-1, the viral replication machinery includes a DNA polymerase (UL30), a DNA polymerase processivity factor (UL42), single-stranded DNA-binding protein (ICP8), helicase–primase complex (UL5, UL8, UL52), and an origin binding protein (UL9) [61,62]. Replication proteins encoded by VACV include a DNA polymerase holoenzyme that consists of a DNA polymerase (E9), a processivity factor (A20 and D4 heterodimer), an ATPase/primase (D5), a single-stranded DNA-binding protein (I3 SSB), a scaffold protein (H5), a DNA ligase (A50), a FEN1-like nuclease (G5), and a Holliday junction resolvase (A22) [63]. By iPOND, the core components of the viral replication machinery were found to be associated with replicating the DNA genome of each virus, and cellular DNA polymerases were almost completely absent [30,31,33]. These data are further evidence that replicating viral DNA rather than replicating cellular DNA was purified in these viral iPOND assays.

In addition to virally encoded replication factors, many host factors were found to be associated with replicating viral DNA. One cellular replication protein that was found to be associated with replicating genomes across all three viruses is the proliferating cell nuclear antigen (PCNA). In uninfected cells, PCNA is the processivity factor that encircles DNA and tethers cellular DNA polymerases to the replicating DNA. PCNA also acts as a scaffold to tether DNA damage, repair, and chromatin remodeling factors to replicating DNA while also playing a role in Okazaki fragment maturation and translesion synthesis [64]. PCNA binds to HSV-1, VACV, and Ad5 DNA during viral DNA replication [30,31,33]. Components of the sliding clamp loader, replication protein C (RFC 1–5), were also found to be associated with replicating HSV-1 DNA, thus suggesting a mechanism by which PCNA is loaded onto viral DNA [30]. Additionally, separate studies have shown that PCNA knockdown by siRNAs and treatment with PCNA inhibitors reduced HSV-1 DNA replication and late gene expression [65,66]. By iPOND, it was shown that PCNA inhibition can block DNA polymerase (UL30) and DNA repair protein binding to viral DNA, including the HSV-1 uracil DNA glycosylase (UL2) [66]. During VACV infection, PCNA has been identified to interact with the VACV polymerase E9 and be recruited to sites of viral DNA replication in the cytoplasm [67]. Additionally, chemical inhibition of PCNA blocked genome replication and late gene expression, and RNAi-targeting PCNA blocked genome replication and E9 recruitment to viral DNA. Despite encoding their own viral processivity factors (UL42 for HSV-1 and A20:D4 heterodimer for VAVC), PCNA may facilitate roles in HSV-1 and VACV infection that viral proteins are unable to perform.

Topoisomerases were also identified as cellular replication proteins that associate with the genomes of these DNA viruses [30,31,33]. Three main cellular topoisomerases were identified, including Top1, Top2A, and Top2B. Top2A and Top2B are type II topoisomerases that function to relax supercoiled DNA by creating double-strand breaks (DSBs), while Top1 is a type I topoisomerase that creates a nick in a single strand of DNA [68]. Type II topoisomerases are important for DNA replication, and type I topoisomerases are important for DNA replication and transcription. It makes sense that these viruses would utilize cellular topoisomerases since VACV does not encode a type II topoisomerase, and HSV-1 and AdV do not encode either type I or II topoisomerases. For VACV, immunofluorescence microscopy has confirmed that Top2A and Top2B localize to the nucleus in uninfected cells [69]. However, during VACV infection, topoisomerases are evenly dispersed between the cytoplasm and nucleus. For HSV-1, Top1 has been found to copurify with the viral genomes through the entire infection process, whereas Top2A and Top2B only copurify with replicating viral DNA after the onset of DNA replication [34]. Regarding Ad5 replication, there is an accumulation of Top1 at sites of viral DNA synthesis [31]. Consistently, Top1 and Top2 cleavage of AdV DNA is required for DNA replication [70]. It is likely that type I and II topoisomerases play similar roles in processes that occur on viral DNA as they do in processes that occur on cellular DNA by relieving topological stress induced by the unwinding of double-stranded DNA.

### 4.3. DNA Damage Response (DDR) and Repair Proteins

The DNA damage response (DDR) is a well-orchestrated signaling pathway that recognizes and responds to DNA damage and protects cells from genomic instability (reviewed in [71]). The DDR is driven by proteins of the phosphatidylinositol 3-kinase-like protein kinases (PIKKs) family: ataxia-telangiectasia mutated (ATM), ataxia-telangiectasia-and-Rad3-related (ATR), and DNA-dependent protein kinase (DNA-PK). The ATM and DNA-PK kinases are activated in response to double-stranded breaks (DSBs), while ATR responds to single-stranded DNA (ssDNA) generated at sites of stalled replication [71]. Upon sensing DNA damage, ATM/ATR phosphorylates a myriad of substrates, which further recruit downstream proteins to mediate repair and restore the damage. In addition, cells have evolved a number of DNA repair pathways to repair specific types of DNA lesions. For example, small chemically modified bases are repaired by base excision repair (BER), and mispaired DNA bases are removed by the actions of the mismatch repair (MMR) pathway. Bulky lesions like pyrimidine dimers or intrastrand crosslinks (ICLs) are repaired by nucleotide excision repair (NER) or the Fanconi anemia (FA) pathway. Finally, DSBs are processed by either nonhomologous end-joining (NHEJ) or homologous recombination (HR), while single-stranded breaks (SSBs) are repaired by the single-strand break repair (SSBR) pathway.

It has been established in multiple systems that viruses manipulate DDR pathways to facilitate their own replication [61,72,73,74,75,76]. While several DNA repair proteins are degraded through viral-encoded E3 ubiquitin ligase activity, others are co-opted for productive infection [77]. Furthermore, most viruses differ in the way they interact and coordinate with the host DDR. For instance, the Mre11/Rad50/Nbs1 (MRN) complex and ATM are activated by the HSV-1 genome and act to stimulate its replication [78]. In contrast, the AdV proteins E4orf6/E1b55K and E4orf3 prevent MRN activity by degradation and relocalization of the complex away from viral replication compartments [79].

Cellular DDR proteins interact with infecting or actively replicating viral DNA throughout the viral infectious cycle. By labeling actively replicating HSV-1 DNA with EdU or EdC and performing iPOND or aniPOND, several components of double-strand break repair (DSBR), MMR, and BER pathways were found to be enriched on replicating genomes [30,32]. One of the identified components was Ku70 (or XRCC6), a subunit of DNA-PK that binds DSBs and mediates repair via the NHEJ pathway. The Ku70 protein was also found to localize to HSV-1 replication compartments [30] and inhibit HSV-1 DNA replication [12]. It was speculated that Ku70 plays an antiviral role by binding the viral genome and restricting infection. Consistent with this hypothesis, it was recently shown that the DNA sensor IFI16 recruits DNA-PK to HSV-1 DNA early during infection. This results in an antiviral response that suppresses viral mRNA and protein production while stimulating antiviral cytokines, including interleukin-6 (IL-6) [80].

Proteins of the MMR pathway (MSH2, MSH3, MSH6, and MLH1) were identified to be among the most abundant repair proteins enriched on replicating HSV-1 DNA by MS [32]. In addition, immunofluorescence studies showed robust colocalization of MLH1, MSH2, and MSH6 at viral replication compartments at 4 hpi. It is interesting to note that the MMR proteins were recruited in a DNA synthesis-dependent manner and were absent from HSV-1 pre-replication sites [32,34], suggesting a role in replication-coupled repair of HSV-1 DNA. Previously, MSH6 was found to interact with the HSV-1 single-stranded DNA-binding protein ICP8, while MSH2 and MLH1 were required for efficient HSV-1 infection [81]. This raises the possibility that incorrect bases inserted by the HSV-1 DNA polymerase might be replaced by cellular MMR proteins to maintain high fidelity of viral genomes [82]. However, the functional significance of MMR during HSV-1 DNA replication has not yet been defined.

In the comparative study of HSV-1, Ad5, and VACV by Reyes et al., they identified the DNA repair proteins ERCCL6 and SLX4 at viral replication compartments of all three viruses [31]. Both these proteins stimulated HSV-1 and Ad5 DNA accumulation and protein production. These data suggest that viral genomes contain DNA lesions that need to be resolved during the viral infectious cycle. However, it is unclear how and when these lesions are generated on the viral DNA.

Dembowski and DeLuca utilized stable isotope labeling of amino acids in cell culture (SILAC) during HSV-1 infection to distinguish between proteins that enter the nucleus along with the infecting virion and proteins that are synthesized within the infected cell [34]. Using the SILAC-MS analysis, they determined specific cellular DNA repair proteins that are associated with viral genomes at distinct stages of infection. At 1 hpi, SSBR proteins PARP1, PARP14, RPA1, and DNA ligase 3 (LIG3) were recruited to HSV-1 DNA and remained associated throughout replication. In contrast, cellular proteins involved in DSBR, MMR, and BER pathways were recruited during DNA replication. Proteins recruited early in infection could potentially be immediate response factors that detect the incoming viral genome as damage. An alternative hypothesis could be that SSBR proteins play a protective role by binding specific regions of the viral genome, preventing recognition by other cellular factors. The FA pathway is another DNA repair activity stimulated by HSV-1 infection and required for productive viral replication [83]. Components of the FA pathway interact with viral replication proteins and were found within viral replication compartments during replication. Further investigation is required to assess the functional significance of the temporal regulation of DNA damage proteins by viruses.

Factors that accumulate on viral DNA may have multifaceted roles in processes important for viral infection. The poly(ADP-ribose) polymerase (PARP) family of enzymes maintains the post-translational modification (PTM)-termed poly(ADP-ribose) (PAR) on itself and on multiple target proteins. PARPs participate in a variety of cellular functions, including chromatin remodeling, DNA repair of DSBs and SSBs, stabilization of replication forks, and processing of unligated Okazaki fragments during replication [84,85]. Other members of the PARP family have been implicated in the antiviral innate immune response [86]. HSV-1 infection can activate PARP1 by inducing PAR formation during viral replication [87]. PAR chains are subsequently removed by the enzyme poly(ADP-ribose) glycohydrolase (PARG). Interestingly, HSV-1 E3 ligase protein ICP0 mediates the degradation of PARG specifically late in infection, although early inhibition by siRNA was inhibitory to infection. Gammaherpesviruses, including EBV and KSHV, have also been shown to regulate PARP1 during infection [88]. Additionally, PARP1 is enriched on HCMV and VACV genomes [33,41], although there is no significant evidence yet for its role in their replication. These viral systems could provide attractive models for studying the functions of PARPs on DNA during infection and potentially identify novel cellular pathways.

### 4.4. Antiviral Restriction Factors

Factors known to contribute to viral repression are directly associated with incoming viral DNA genomes. These include cellular proteins associated with promyelocytic leukemia nuclear bodies (PML NBs; also known as nuclear domain 10 or ND10) [89]. PML NBs are multi-protein complexes with the key protein PML acting as a scaffold and the inner core containing a multitude of transiently associated proteins [90]. PML undergoes SUMOylation, a PTM that drives the recruitment of associated core proteins through their SUMO-interacting motif (SIM). Early in HSV-1 infection, it was found by aniPOND that components of PML NBs (PML, SP100, SUMO2) are enriched on viral genomes but are no longer detected by 2 hpi [34] as a result of the E3 ubiquitin ligase activity of ICP0. This study is consistent with previously published data that incoming genomes are juxtaposed to PML NBs [91,92]. PML bodies, as well as components of the innate immune response (IFIT1, IFIT2, IFI16, TRIM25, and G3BP), were also detected during HCMV infection [41]. The effects of PML on viral DNA are unclear, although evidence suggests a beneficial role in facilitating the initiation of HSV-1 and AdV replication [93,94].

DNA repair factors can also limit viral replication. Incoming viral genomes of HSV-1 stimulate a DNA damage response and recruitment of DNA repair proteins to sites of viral genome deposited in the nucleus, independently of ND10 factors [95]. This response is coordinated by cellular E3 ligase RNF8 and RNF168 but can be counteracted by ICP0, which leads to their degradation [96,97]. The DNA damage marker γH2AX is found at these sites, but it is unclear whether the H2AX histone variant is associated entirely with the viral genome or also the resident host genome. Using iPOND to find proteins that accumulate on HSV-1 genomes in the absence of the ICP0 ubiquitin ligase, Kim et al. isolated known sensors and repressors of the virus, including PML, DNA-PKcs, and IFI16 [37]. This analysis also discovered new restrictions that are counteracted through degradation by ICP0, such as SLFN5. Therefore, identifying cellular proteins associated with viral genomes in the absence of a specific effector is an attractive way to identify unknown antiviral restriction factors.

## 5. Accumulation of Host Proteins to Specific Sub-Compartments during Infection

Viral genomes form specific cellular structures known as viral replication compartments (VRCs) that form hubs for viral processes such as transcription, replication, progeny assembly, and packaging. The initial stages of infection are defined by the formation of pre-replicative foci (Figure 2C), followed by the development of larger and more defined structures (Figure 2A,B) [98]. Visualizing the location of host proteins associated with viral genomes is a complementary approach to iPOND in assessing spatial interactions with viral genomes and can help to determine how these proteins impact virus infection.

### 5.1. Accumulation of Activated ATM within Viral Replication Compartments (VRCs)

DNA viruses have complex intersections with cellular DDR pathways, which can be inhibitory or can be harnessed for virus infection. Consistent with iPOND analyses, several DDR proteins have been shown to accumulate at VRCs. In HeLa cells, activation of the ATM kinase in response to HSV-1 infection was observed by immunofluorescence and immunoblotting analysis with an antibody to the auto-phosphorylated form [78]. Activated ATM, along with DNA repair proteins Rad50 and Nbs1, were observed to accumulate in VRCs [78,99]. Furthermore, ATM activation was an early event that occurred independently of ICP0 expression since the ICP0-deficient virus was still able to activate ATM autophosphorylation. Since the MRN complex recruits ATM to the damage site [100], this study used A-TLD1 patient cell lines expressing a truncated form of Mre11 to demonstrate that complete activation of ATM during infection was dependent on Mre11. Another study demonstrated that while Mre11 restricts replication of an ICP0-null virus, ATM and p53 promote replication of this mutant, and ICP0 expression eliminates these DDR effects [76]. Strikingly, AdV has a much more complex serotype-specific interaction with MRN and ATM, suggesting that viruses have evolved different strategies to overcome the host DDR during infection [101]. A more recent study utilized human corneal epithelial cell lines to show that HSV-1-mediated ATM activation occurred in an ICP4-dependent manner even in the absence of damaged viral DNA [102]. This suggests that the viral entry and interaction of ICP4 with the viral genome was sufficient to trigger DDR. The molecular mechanisms underlying ICP4-mediated ATM activation, however, still need to be elucidated. Polyomaviruses also activate ATM signaling, and the phosphorylated kinase accumulates at VRCs [103,104,105]. It remains to be determined exactly what the consequences are for ATM activation during virus infections and how kinase substrates impact viral DNA replication.

### 5.2. The Role of ATR Signaling during Viral Infection

Initial studies from the Weller lab suggested an uncoupling of ATR and its interacting partner ATRIP during HSV-1 infection [106]. At replication forks, a downstream effect of ATR activation is the phosphorylation of RPA (pRPA) on exposed single-stranded DNA (ssDNA). Using immunofluorescence, the localization of ATR, ATRIP, and pRPA was observed during HSV-1 infection. While ATR did not accumulate in VRCs, ATRIP and a subset of pRPA were observed to colocalize with the heat shock protein Hsc70 in virus-induced chaperone-enriched (VICE) domains. VICE domains are comprised of heat shock proteins and other cellular protein quality control components, including ubiquitin and proteosome subunits, and are implicated in the sequestration and degradation of misfolded viral proteins [107]. It was concluded that HSV-1 dismantles ATR signaling by relocalizing ATR interacting partners ATRIP and RPA to VICE domains. In a subsequent study, Mohni et al. used more specific antibodies to show that ATRIP and RPA colocalize with ICP8 in VRCs, but pRPA does not, suggesting that downstream ATR signaling is not activated [108]. This was further validated using a kinase-dead ATR, which also accumulated at VRCs, confirming that ATR localization to VRCs was independent of its kinase activity. Loss of ATRIP led to a slight delay in the expression of immediate-early and early genes and a five-fold reduction in progeny production, indicating that while ATR signaling is not absolutely required for productive HSV-1 infection, it does contribute to efficient infection probably by stimulating the expression of IE genes. Additionally, other proteins involved in ATR signaling are also recruited to HSV-1 VRCs, including Rad9, Hus1, Rad1, Rad17, TopBP1, CINP, and Chk1, although phosphorylation of Chk1 is absent [109]. This further suggests that the ATR signaling pathway is not active during HSV-1 infection. Most of these ATR signaling proteins recruited were required for efficient HSV-1 replication since viral yields were lower when these proteins were knocked down. Interestingly, when ATR was activated prior to infection, a small but significant two-fold reduction was observed in recombination frequencies between coinfecting genomes [109]. Another contradicting study demonstrated the formation of nuclear and cytoplasmic foci of pATR and pChk1 during HSV-1 infection [110]. Using immunofluorescence, proximity ligation, and cellular fractionation, significant colocalization of pATR and pChk1 with ICP4 and ICP0 in the cytoplasm was detected in this study. However, no further examination of the nature of these cytoplasmic foci was performed, and therefore, the consequences of these interactions are unclear.

### 5.3. Association of PML NBs with Incoming Genomes

To examine systematically the recruitment of host repair proteins to VRCs, HSV-1 infection has been categorized into distinct stages. Incoming HSV-1 genomes are associated with ND10 (or PML NBs), which are characterized by the PML protein [89]. Disruption of ND10 by ICP0 correlates with the formation of pre-replicative sites lacking the viral DNA polymerase [111]. Recruitment of the polymerase to replication sites is characterized by the redistribution of certain isoforms of PML to replication centers [112]. Homologous recombination (HR) proteins, RPA, RAD51, and Nbs1, are all predominantly recruited to pre-replicative sites only after ICP0-mediated disruption of ND10 and when the viral polymerase is present [113]. These data suggest that disruption of ND10 is essential to gain access to these proteins for efficient viral replication. So far, there is no evidence that HR proteins are directly associated with PML NBs during infection, and more mechanistic approaches are required to study this interplay. Their association and function directly on viral genomes are yet to be fully understood.

### 5.4. Selective Recruitment of Key Regulators of DDR

HSV-1 manipulates the host DDR to inactivate the antiviral components and utilize other factors for optimal replication [76]. HSV-1 mediates the degradation of key host E3 ubiquitin ligases, RNF8 and RNF168, that are involved in DSBR [96]. RNF8 and RNF168 proteins belong to the RING domain-containing E3 ubiquitin ligase family and promote ubiquitination and recruitment of downstream DSB proteins, including 53BP1 and BRCA1, at distinct stages of DSBR [114]. p53-binding protein 1 (53BP1) and BRCA1 maintain genome integrity by regulating NHEJ and HR, respectively [115]; however, their role during viral infection is unclear. A weak recruitment of BRCA1 to HSV-1 VRCs and a much stronger 53BP1 accumulation were shown by two independent studies [95,116]. Recruitment of 53BP1 was RNF8- and RNF168-dependent but independent of Mre11 and ATM [95]. The order of events in this signaling cascade is similar to the host DDR signaling at DSBs.

Interestingly, the CCCTC-binding factor (CTCF) accumulated in VRCs in an ATM-dependent manner [116]. CTCF is a highly conserved zinc finger protein that interacts with a consensus sequence on the DNA, with known functions in transcriptional regulation and genome organization [117]. Additionally, new roles of CTCF have emerged in facilitating the formation of γH2A.X domains during DSBR [117]. Whether CTCF participates in host DDR downstream of ATM still needs to be investigated.

### 5.5. Dynamic Interactions of RNA Polymerase II with Replicating Viruses

During infection, HSV-1 and AdV genes are transcribed by the host Pol II. To visualize the interaction of Pol II at HSV-1 VRCs, cells were co-stained with antibodies specific to Pol II, phosphorylated Pol II (Ser2P or Ser5P), and ICP4 [116]. Pol II phosphorylation marks active transcription and transition from initiation to elongation [118]. While total Pol II colocalized with ICP4, the phosphorylated forms only accumulated in smaller VRCs, and the signal was less intense in fully formed larger VRCs [116]. These observations suggest that active transcription is more prevalent in smaller VRCs rather than in larger fused VRCs.

## 6. Three-dimensional Genome Techniques to Evaluate Interactions during Viral Infection

Studies investigating DNA–protein interactions during viral infection have mostly addressed the proteins that are associated with viral genomes. However, manipulating protein interactions with cellular chromatin is equally important for the outcome of infection. The cellular genome is organized into a complex three-dimensional conformation that plays a crucial role in regulating fundamental processes, including transcription and replication. The advancement of 3D chromatin capture technologies has provided us with extraordinary knowledge of how chromatin is organized and packaged into the nuclear environment [119,120]. The original chromosome conformation capture (3C) experiments by Dekker and colleagues revealed that chromatin is organized in a series of structurally folded domains called topologically associating domains (TADs). Genome organization regulates cellular processes by bringing distant loci into close physical proximity through chromatin looping [121]. These loops demarcate the TADs and are bound by CTCF and the cohesin subunits RAD21 and SMC3 [122]. DNA viruses must navigate the complex host chromatin architecture in order to support their own gene expression and replication. 3D chromatin capture technologies have enabled the study of virus–host interactions at the level of higher-order chromatin structure, reviewed in [123].

Using Hi-C and viral DNA capture (Chi-C) on primary human hepatocytes infected by Hepatitis B virus (HBV) or Ad5, it was shown that viral DNA preferentially interacts with active chromatin [124]. Specifically, HBV interacts with CpG islands upstream of active genes and is enriched for cellular factor CXXC finger protein 1 (Cpf1). Cpf1 binds to non-methylated CpG islands to maintain an active chromatin state. Cpf1 is bound directly to HBV covalently closed circular DNA (cccDNA), and the depletion of Cpf1 reduces HBV RNA, suggesting that Cpf1 is required for HBV transcription. Additionally, this study also demonstrated that Ad5 preferentially interacted with transcription start sites (TSSs) and enhancers of highly expressed genes during infection. The implications of these interactions are yet to be defined.

During infection, HSV-1 significantly interacts with and remodels specific nuclear structures (e.g., PML NBs, centromeres, and telomeres) [125,126,127]. HSV-1 single-stranded DNA-binding protein ICP8 colocalizes with telomeric proteins and promotes viral replication. Furthermore, HSV-1 induces transcription of telomere repeat-containing RNA (TERRA). It is interesting to note that repressed latent HSV-1 genomes associated with centromeres and ICP0-mediated proteasomal degradation of centromeric proteins (CENP-A, CENP-B, and CENP-C) destabilize the centromeric domains. While the underlying mechanisms are not well understood, one explanation could be that cellular proteins enriched at these nuclear structures somehow benefit viral propagation and, therefore, associating with these structures provides the virus with easy access to proviral factors. Consistent with this, it was found that KSHV infection upregulated homologous recombination-dependent telomere elongation (alternative lengthening of telomeres), which was essential for the establishment of viral latency [128]. The expansion of viral replication centers may not be random, and viral genomes likely establish specific contacts with host chromatin during infection. The observation that TAD demarcation protein CTCF accumulates at HSV-1 VRCs [116] suggests that HSV-1 might organize itself into TAD-like structures. By applying 3D genome techniques to viral infection, we could begin to answer some outstanding questions in the field, such as how the host cellular chromatin structure benefit infection. How is the cellular chromatin regulated by viruses? And finally, how is the viral genome organized within the complex milieu of the host nucleus?

## 7. Future Outlook

The association of proteins with viral and host genomes during infection governs all aspects of what happens inside infected cells and will ultimately determine the outcome of infection. Interactions mediate transcription of viral and host genomes, replication and packaging of viral genomes, and host defenses that control infection. The development of technologies such as iPOND has helped identify a vast repertoire of host proteins that interact with viral DNA during infection. Additionally, these studies highlight how various viral processes, such as replication and transcription, might be tightly coupled, both spatially and temporally. However, huge gaps in knowledge remain. Key aspects of how these interactions control infection require careful validation and investigation. Insights into how they govern the infectious cycle of viruses can be revealed by addressing the following questions: (1) What is the functional significance of the identified host proteins in regulating viral replication and viral spread? (2) What are the specific factors that mediate the recruitment of host factors to viral genomes? (3) How does the spatial organization of replicating viral genomes regulate viral processes? (4) How do specific temporal interactions coordinate the different stages of infection? (5) And how do contacts of viral proteins and genomes with the host chromatin change during the infectious cycle to impact viral infection? This last question is particularly interesting as it helps determine how the chromatin state of the infected cell impacts viral infection. Performing these proteomic techniques with viral mutants will reveal cellular restriction factors that are intentionally kept away from viral genomes, as well as cellular transcription and replication factors that are specifically recruited through interactions with viral DNA-binding proteins. Further studies that incorporate iPOND throughout the infectious cycle, combined with pulse-chase labeling of replicating genomes, will reveal the distinct factors recruited at different stages of the replication process. Improvements in the sensitivity of mass spectrometry and new labeling approaches will provide greater insights into distinct steps of infection. Developing ways to label viral genomes in live cells will capture the dynamics of interactions and the fate of newly synthesized viral genomes within infected nuclei. In this review, we have focused on cellular factors but have not addressed histones and their modifications found in viral genomes. This epigenetic control of infection has been reviewed elsewhere [129,130,131,132,133]. The proteins identified by the proteomic approaches we describe clearly impact viral chromatin, persistence of viral episomes, association with cellular chromatin, and maintenance of histone modifications. Understanding how protein–DNA interactions allow viruses to navigate the complex environment of the host nucleus during infection will continue to reveal novel aspects of viral pathogenesis as well as host cellular processes. In addition, antivirals could be developed that block host protein recruitment to viral DNA or host protein function on viral DNA, although cytotoxic effects would have to be considered.
